# A Multivariate Analysis-Driven Workflow to Tackle Uncertainties in Miniaturized NIR Data

**DOI:** 10.3390/molecules28247999

**Published:** 2023-12-07

**Authors:** Giulia Gorla, Paolo Taborelli, Barbara Giussani

**Affiliations:** Department of Science and High Technology, University of Insubria, Via Valleggio 9, 22100 Como, Italy

**Keywords:** data uncertainty, miniaturized NIR, ASCA, multivariate error, image analysis

## Abstract

This study focuses on exploring and understanding measurement errors in analytical procedures involving miniaturized near-infrared instruments. Despite recent spreading in different application fields, there remains a lack of emphasis on the accuracy and reliability of these devices, which is a critical concern for accurate scientific outcomes. The study investigates multivariate measurement errors, revealing their complex nature and the influence that preprocessing techniques can have. The research introduces a possible workflow for practical error analysis in experiments involving diverse samples and instruments. Notably, it investigates how sample characteristics impact errors in the case of solid pills and tablets, typical pharmaceutical samples. ASCA was used for understanding critical instrumental factors and the potential and limitations of the method in the current application were discussed. The joint interpretation of multivariate error matrices and their resume through image histograms and K index are discussed in order to evaluate the impact of common preprocessing methods and to assess their influence on signals.

## 1. Introduction

Analytical measurements are inherently prone to errors, leading to uncertainty in derived outcomes: measurement uncertainty concerns all measurements, regardless of instruments and methods used. Understanding measurement errors and their impact on results is a crucial aspect as only by monitoring them can uncertainty be effectively reduced [1,2].

Recent developments in miniaturized near-infrared (NIR) instruments have shown their effectiveness in diverse applications [3,4]. Literature is witnessing an increase in evidence supporting the utility of these instruments. The affordability and user-friendly interfaces of these sensors have led to a rise in end-users seeking guidance on suitable acquisition strategies [5]. Despite the emphasis on proper sampling and analytical procedure optimization, limited attention has been given to the nature of measurement errors inherent in raw data [6].

Data collected from portable NIR sensors frequently undergo multivariate data analysis, aiming to extract valuable information from noise. Often, techniques developed to analyze multivariate data make simplistic assumptions about measurement errors and its homoscedasticity, uncorrelation, and normal distribution. However, the reality is that the structure of multivariate measurement errors can be much more complex. Many preprocessing techniques applied to multivariate data, such as scaling and derivative filtering, aim to implicitly align the errors closer to the independent and identically distributed (iid) normal pattern [7].

Studying the nature of multivariate measurement errors offers several pivotal benefits. Firstly, it allows measurement quality to be improved by addressing sources of error that restrict precision. Secondly, knowledge of error characteristics allows the design of data analysis tools optimized to handle errors efficiently, leading to more effective extraction of chemical information. For instance, this knowledge can lead to the development of tailored signal preprocessing techniques or better application of existing ones [2,8,9,10].

Finally, the inherent error structure in multivariate data can propagate through different preprocessing and data analysis steps, determining its impact on the result. This comprehensive understanding is vital for accurate, reliable, and meaningful analysis in various analytical contexts [11].

This study proposes a workflow that can be followed to gain insights into errors. Experiments were conducted with different samples and different instruments or the same instrument using different accessories in real working conditions that might occur in research or industrial settings. The primary objective is not the direct comparison of the instruments under study to pinpoint the best sensor. Instead, the aim is to emphasize that varying errors in the data may arise depending on the sample and the instruments used. The research proposes a statistical methodology to unveil the limitations of these tools by providing a framework for their investigation. For each spectrometer, an analysis of its characteristics is undertaken to examine the reproducibility of data and gather insights for optimization.

In previous studies, it has been demonstrated how error estimation through replicates can be valuable, and the information obtained can be used to optimize acquisition conditions, facilitate calibration transfer [10,12], and guide the selection of signal preprocessing techniques [13]. In addition, it has been proven how several factors influence the spectra of miniaturized NIR instruments and, consequently, the associated errors [14,15].

Compared with previous investigation on loose samples from food industries [6], this research aims to verify that the same principles apply to solid samples typical in pharma industries. Spectra of samples belonging to pharmaceuticals and supplementary diets field were acquired. The interest in these samples arise from their physical and chemical characteristics of compactness, shape, color, and components, which are common to many pharmaceutical products, but not only.

The possibility of employing Analysis of Variance (ANOVA) Simultaneous Component Analysis (ASCA) to guide the selection of replicates for studying errors under possible real-world conditions was explored. The influence on the spectra of acquisition condition was investigated. The potentials and limitations of ASCA for this type of study are presented. Furthermore, the study proposes a combination of graphical and numerical methods to assess the influence of preprocessing methods on the multivariate errors. Some common preprocessing methods and their influence on the errors structures are investigated. The trend of a simple correlation index and a visual and graphical method are presented to investigate and compare the efficiency of signal preprocessing in different case scenarios: different samples, miniaturized instruments, and experimental conditions.

## 2. Results and Discussion

### 2.1. Spectral Data and Reproducibility

**Figure 1:**
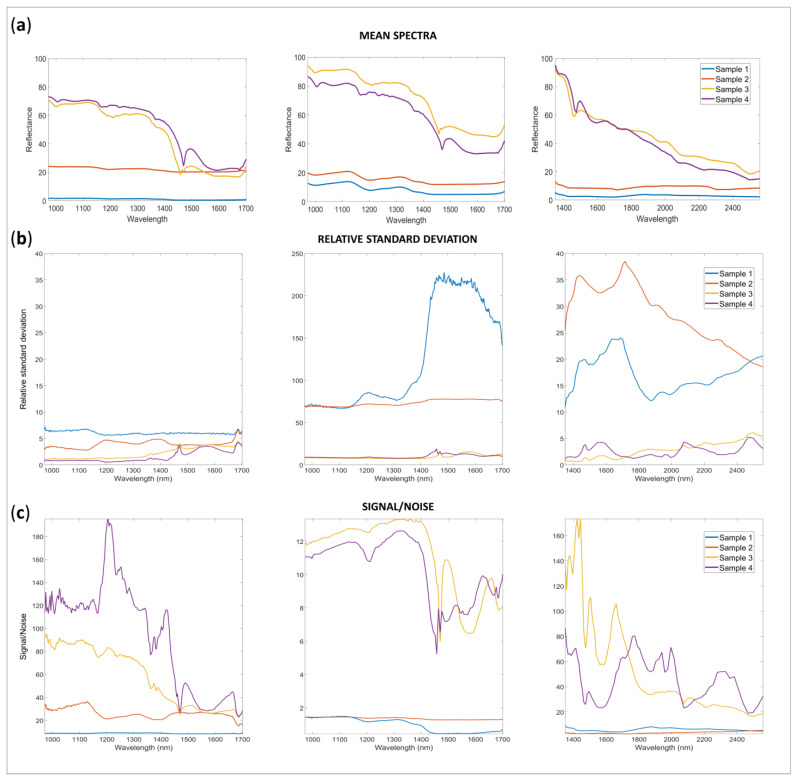
Mean spectra (**a**), relative standard deviation (**b**) and signal to noise ratio (**c**) for the spectra of all samples acquired under charge (90 experimental replicates) from left to right: AvaSpec-Mini-Nir Integrating sphere; AvaSpec-Mini-Nir Optical fiber; and NeoSpectra Scanner.

Data were organized in matrices according to instrumentation and configuration of acquisition. Figure 1 represents the mean spectra obtained from all the replicates acquired with the instruments under charge (90 experimental replicates per sample per instrument). The spectrometers cover ranges in different NIR regions [16]: AvaSpec-Mini-NIR data are in Region 1 (800–1200 nm) and Region 2 (1200–1800 nm), while NeoSpectra spectra are in Region 2 and Region 3 (1800–2600 nm), meaning that different overtone and combinations bands could be seen. In accordance with the technological characteristics of the two spectrometers, such as spectroscopic range, acquisition window, and acquisition configuration, the spectra obtained for the samples are quite different in shape and data quality characteristics (intensity, S/N, reproducibility). It is interesting to notice that the spectra obtained by changing the accessory equipped to AvaSpec-Mini-NIR resulted in similar shapes but substantially different data quality characteristics. Indeed, as expected, the spectra obtained with the integrating sphere are more defined and have higher intensity than those obtained with the optical fiber. For AvaSpec-Mini-NIR, the integrating sphere allows for a more intense recovery of the light reflected, and the sample could be positioned in the internal part of the sphere hole, being invested from the light sources. The optical fiber has a reduced window for the light to pass through, and the sample could be covered only punctually. In addition, in the first case, all the light scattered is recovered, while in the second, part of the light is probably dispersed at the sample level and through the passage into the fiber. Although the spectrometer and detector are the same, differences in light source, light path through the fiber or sphere, and the exposed sample portion to the acquisition windows contributed to these variations.

Generally, for all the configurations and spectrometers, the spectra obtained for Sample 1 and Sample 2 are less reflective than those obtained for Sample 3 and Sample 4. These results could be explained by considering the different colors and opacity of the sample, as well as the different shapes: indeed, Sample 1 and 2 are curved pills while Sample 3 and 4 are plain, almost white tablets.

An interesting observation is that the mean reflectance for Sample 2 is substantially higher with all the instruments with respect to Sample 1: using the AvaSpec-Mini-NIR with optical fiber, the spectra shapes of Sample 1 could even be challenging to identify. The different results obtained for the different configurations or spectrometers could be easily understood if the nature of the measurement is considered. Indeed, according to the configuration of acquisitions, the window width and positioning allowed for each instrument or configuration used to acquire the spectra are quite different (Supplementary Figure S1).

Figure 1c represents the relative standard deviation and S/N ratio for all the samples and spectra acquired. The S/N was calculated as the ratio between the mean spectra for each sample and the standard deviation of the spectra of the same sample. The relative standard deviation was estimated as 100 × standard deviation of spectra (90 replicates) for each sample divided by the mean spectrum for the same sample. In Tables S1 and S2 of Supplementary Materials, other descriptive statistics are summed up. On the whole, spectra acquired using the integrating sphere have the highest reproducibility (lower RSD) and S/N ratio.

### 2.2. Study of the Variability Sources in the Data

ASCA models were calculated for each dataset divided according to the sample. The main experimental conditions were considered as possible important factors. Their interactions were also studied. The influence of signal preprocessing was investigated. The results of ASCA models for AvaSpec-Mini-NIR on each sample with and without signal preprocessing are summarized in Table 1 and Table 2. The percentage each effect contributes to the sum of squares is reported and significant effects are highlighted in bold.

Since ASCA uses ANOVA there are some typical assumptions to consider when interpreting the results. The observations are assumed to be independent of each other, the data come from normally distributed populations with equal variances and the effects of different factors are assumed to be additive. ASCA also uses Principal Component Analysis (PCA), which assumes linear relationships between variables. At first glance and confirming the results obtained in a previous work [15], the outcomes of the models could be interpreted as follows: some factors showed significance in different models (data in bold), while others did not, making it possible to establish their relationship with a specific spectrometer and/or the characteristics of the sample. In general, the effect values were higher after applying preprocessing compared with raw data. Factors significant only for specific samples in raw spectra became significant for all samples under investigation when a scattering correction method was applied.

Comparing the results obtained with the different acquisition configurations for AvaSpec-Mini-NIR, it appears that the significant factors and interactions (data in bold) remain consistent regardless of the configuration and preprocessing method. The slight disparities in significant effects observed in raw data could be due to the inherent variability in data obtained via fiber optic cable compared with the integrating sphere.

The results of ASCA models for the Neospectra Scanner on each sample with and without signal preprocessing are summarized in Table 3. Regarding the other instrument, the results obtained here showed a significant portion of variance left unexplained by the model, and the variances attributed to the factors are lower than the residual variance.

At this point, model results provide available information to identify influencing factors and interactions and describe and understand the experimental conditions. Using these models, it is possible to optimize the data acquisition method and incorporate sources of variability as needed to develop the analytical method. In other words, this information provides insights that one must consider while assessing associated errors. Awareness about the factors affecting the analyses, contingent upon the sample type and that emerge through preprocessing methods, is valuable information for measurement error definition. Indeed, this understanding is crucial for familiarizing with the data, determining the type of error study required, and selecting the appropriate usage conditions. For example, constructing a model in a single session is not feasible or realistic, even if this factor significantly impacts the analyses. Hence, there will be a need to acquire data across multiple sessions, considering the distinct magnitude of errors in each. As for the background condition, knowing its influence allows to consider performing a background analysis before each sample consistently to minimize repeatability issues.

The ASCA model results were assessed by visualizing and interpreting scores and loadings as in typical PCA analysis. From the investigation of the ASCA sub-models, some considerations arose about the importance of visually interpreting the outcomes to gain a comprehensive understanding of the results.

**Figure 2:**
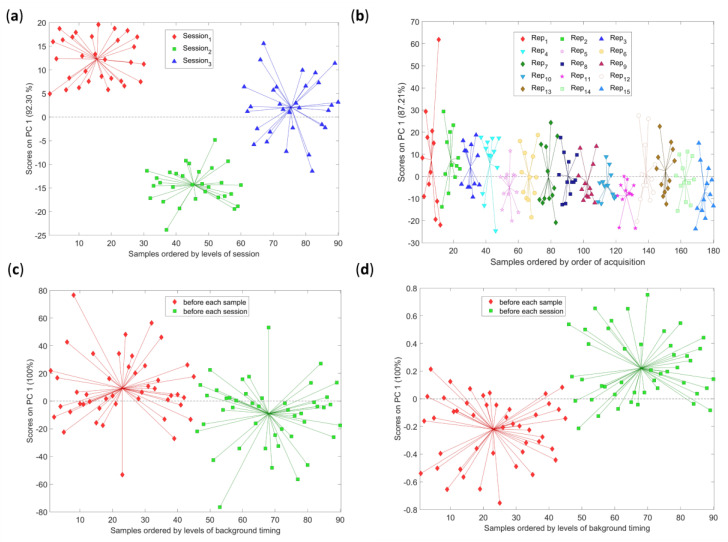
ASCA sub-models examples. Scores of the ASCA sub−model for the factor (**a**) session (**b**) replicates (**c**,**d**) timing of background. Instruments: (**a**) AvaSpec-Mini-NIR equipped with integrating sphere (**b**) NeoSpectra Scanner (**c**,**d**) AvaSpec-Mini-NIR equipped with optical fiber. Samples: (**a**) Sample 3 (**b**) Sample 2 (**c**,**d**) Sample 4.

Figure 2 shows examples to discuss the results obtained and the perspectives achieved. The scores obtained from the sub-models are displayed based on factor levels, and spider graphs are employed to emphasize the scores’ centroids. The loadings for the respective models are shown in Supplementary Figure S2.

The examples represent the following situations: (a) show a sub-model for a significant factor (session) with 42.68% effect; (b) display a sub-model for a non-significant factor (order of replicates) with 4.96% effect; (c) present a non-significant factor (timing of background) with 1.76% effects; and (d) show the exact same sub-model of (c) performed on data preprocessed with standard normal variate (SNV) and resulted in significant factor with 8.44% effect.

By visual inspection, generally, trends and groupings observed in the graphs indicated the significance of the factors: (a) is an example where the grouping is evident and the results of ASCA could be considered reliable. In the case of non-significant factors what could be expected is that the distribution of the spider graphs is random within a distribution around 0. In other words, the scores obtained belong to the same normal population as in the case (b).

A noteworthy feature of ASCA emerges by comparing (c) and (d). A statistically non-significant factor (*p*-value > 0.05 estimated through permutation test) could carry interesting information. The visual inspection of the sub-models resulted in similar loadings and scores for the two case scenarios: raw data and preprocessed. Such factors could be essential to evaluate for future works or studies, and the need for considering several aspects to carry out a conclusion emerged.

**Figure 3:**
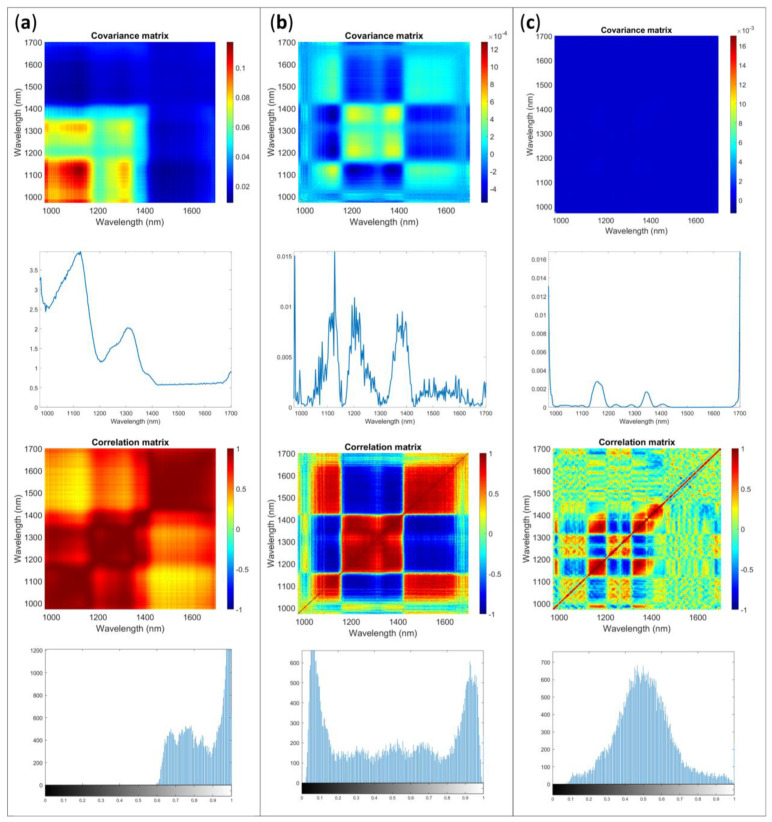
Multivariate error covariance matrix, error covariance matrix diagonal, correlation matrix and image histogram of the correlation matrix for Sample 1 acquired with AvaSpec-Mini NIR with integrating sphere: (**a**) raw data, (**b**) SNV, (**c**) first derivative.

In general, how the quality of spectra influences ASCA results is particularly evident when comparing the outcome for fiber optic and integrating sphere measurements. It is observed that the main significant factors are the same, but their magnitudes differ. The factors have a lower influence on the measurements with less accuracy (fiber optic) and higher influences on the more accurate configuration (integrating sphere). This is not ascribable to the fact that the studied factors do not influence optical fiber measurements but rather because the variability associated with spectra is so substantial that the score distributions in ASCA are very wide and, therefore, not significantly distinct. The same phenomenon occurs in the example in Figure 3 for the background timing, when SNV preprocessing is applied. Spectra exhibit a sharper distribution after scattering removal, allowing for a more evident differentiation between populations with different background time intervals. As a result, it is not advisable to consider only *p*-values: pairing the validation through permutation [17] with visual inspection appears of fundamental importance. To put it differently, verifying the absence of interesting tendencies through groups could be fundamental, especially when the data are poor in quality due to instrumental limitations.

### 2.3. Uncertainty Characteristics: Multivariate Error

At this stage, the factors have been identified and characterized, so strategies to calculate the multivariate error in a realistic situation thoughtfully could be introduced.

Multivariate errors were calculated as explained in the Materials and Methods section. Variance-covariance and correlation matrices were obtained and interpreted for raw and preprocessed data of each sample.

#### 2.3.1. AvaSpec-Mini-NIR

AvaSpec-Mini-NIR could work only when connected to a computer and power supply. From ASCA results emerged the importance of session and timing of background. In real experiments, it is assumable that just one background procedure would be used: for example, a background would be acquired before each sample. To include the information related to the differences between sessions and in order to obtain a reliable estimation with sufficient replicates, the multivariate error for each sample was calculated by pooling errors calculated on each session according to [18]. An example of the results is shown in Figure 3 for the integrating sphere accessory.

The shape of error covariance matrices obtained on raw data is in accordance with those for NIR spectrometers reported in previous works [1,2,6,13]. The noise contributions identifiable are consistent and differ in magnitude and specific shapes depending on the sample investigated. The image histogram shows that most of the pixels have rather high intensity values, indicating high correlation between channels (wavelengths). It should be considered rather far from the ideal situation, in which the overall shape expected should be a normal distribution with the mean value around value 0.5 of the gray scale.

The application of SNV, that is typically used on spectra to remove scattering contributions, systematically reduce the error variance structure, and change the correlation between channels. It is interesting to note how the changes induced in the error structure by preprocessing reflect in its distribution. In the image histogram a sort of bi-modal distribution is obtained.

Preprocessing data with Savitzky–Golay derivative allows to obtain the best situation for the data within these examples. Indeed, derivative filtering aims to remove the correlations in the noise and make it closer to independent. In Figure 3c, a substantially planar **Σcov** is shown. Observing the diagonal profile, it is clear the need for removal of the spectra edges and when focusing on the inner part of the diagonal, some peaks are identifiable in the variance, but the magnitude is appreciably lower than with other methods. Observing **Σcor** seems that correlations are present even if with more complex structure. The preprocessing did not provide the ideal and perfect situation, heteroscedasticity is an issue, although the errors are not independent, their correlation pattern appears to be more random compared with the correlation pattern observed in the raw data. The image histogram shows an almost symmetrical light tailed distributions shape centered on 0.5.

In Table 4, the results of the K index calculated for each sample and accessory configuration are reported. The imbedded correlation for each sample is 0.826. The interpretation of correlation coefficient, as well as that of image histogram and visual inspection of the error allow to identify the first derivative as the best options for the data under investigation. The proximity of K index to the imbedded value could be used as hint to evaluate the goodness of the preprocessing. Indeed, K indices comparable to those of the imbedded value for an established number of replicates and variable channels mean that the preprocessing allows to correct almost all the correlations dependent on the experiment.

As expected, the value selected for the window width, polynomial and derivative order could affect the shapes of multivariate error and eventually, the results of prediction and classification models [19,20] as well as the interpretability of exploratory results [21]. The K indices and visual methods presented also proved to be sensitive to the influence of derivative parameters. An example is reported in Supplementary Materials Figure S3.

**Figure 4:**
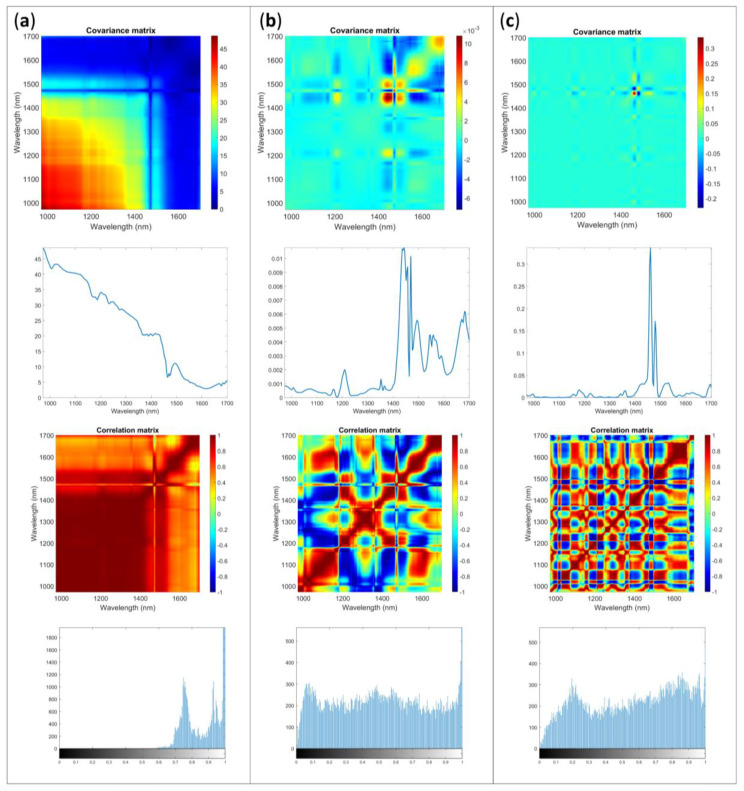
Multivariate error covariance matrix, error covariance matrix diagonal, correlation matrix and image histogram of the correlation matrix for Sample 4 acquired with AvaSpec-Mini NIR with optical fiber: (**a**) raw data, (**b**) SNV, (**c**) first derivative.

Figure 4 shows a case in which a structured error is visible, even after the preprocessing through derivative. Similar results were obtained with both accessories for Sample 3 and Sample 4.

Looking at the covariance matrix it could be expected that after applying the first derivative the error distribution could significantly improve in respect to raw data and SNV preprocess: an almost flat surface is obtained. The correlation matrix appeared more complex and random. However, the K index and the image histogram show a different perspective. The K index value is sufficiently far from the imbedded value for the matrix, suggesting that structured error is present. The image histogram illustrates an asymmetric shape with heavy tail distribution. When investigating the diagonal of **Σcov**, the interpretation is used to identify the structured error that could be attributed to a different content of moisture in the tablets along the subsequent replicate acquisition.

In general, the results after preprocessing showed that different correction are obtainable for different accessory and results more closely to the ideality are achievable when the common preprocessing here considered are applied on spectra acquired with the integrating sphere. The comparison of the results obtained for different samples could be interesting as well. Spectra of Sample 1 and Sample 2 seem to have similar error behaviors when looking at the integrating sphere results for error matrices, correlation index and histogram plots. The slight differences can be mainly related to the scattering influence exercise by the blister. For the same samples, more variance was obtained for the results with the optical fiber.

#### 2.3.2. NeoSpectra Scanner

Concerning the charge condition, NeoSpectra Scanner sensors allow the recording of spectra with the instruments under charge or operating on their own battery. The example reported are those obtained from the use of the spectrometer on its own battery and performing a background before each sample acquisition.

**Figure 5:**
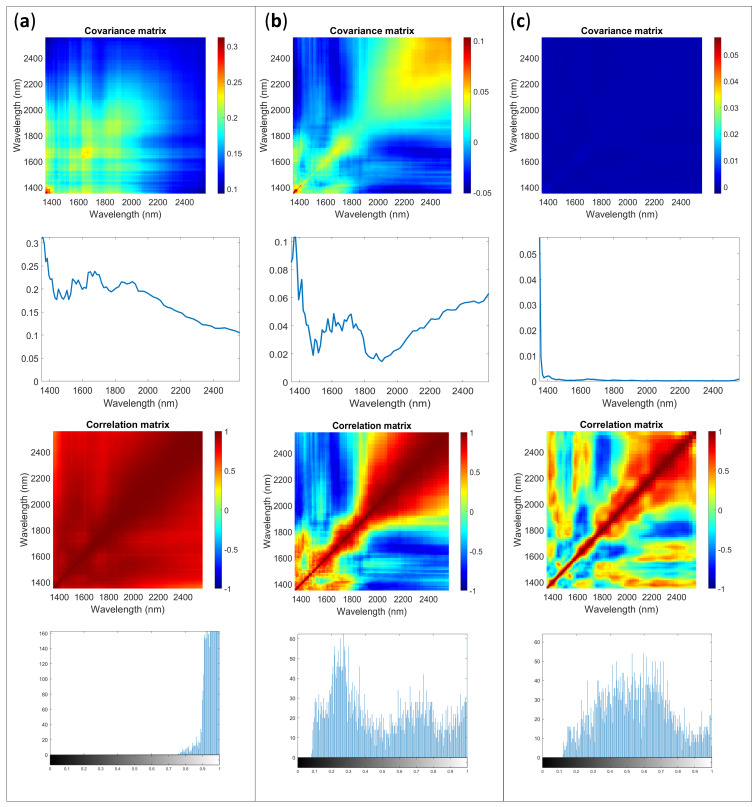
Multivariate error covariance matrix, error covariance matrix diagonal, correlation matrix and image histogram of the correlation matrix for Sample 1 acquired with NeoSpectra Scanner: (**a**) raw data, (**b**) SNV, (**c**) first derivative.

In Table 5 the K index values calculated are shown and in Figure 5 an example of the results obtained is displayed. The imbedded correlation of 0.397 was calculated for 45 replicates and 74 variables.

From the results, it is evident that the error shape, although typical contributions for near-infrared spectra can be identified, significantly differs among spectrophotometers accordingly to the spectral ranges and technologies. Consequently, employing the same preprocessing methods does not yield consistent error modification performance for different instruments. SNV continues to be a method for scattering removal, but in this case, the error contribution for the samples is more complex. The result is characterized by error covariance and correlation surfaces that remain substantially distant from the ideal. This observation is further substantiated by the markedly elevated K index values, exceeding the embedded value. While the error shape and channel correlation certainly improve, they do not approach the ideal, as observed in the case of AvaSpec-Mini-NIR sensor.

Figure 5 shows the case of Sample 1 for NeoSpectra Scanner data.

Different instruments may exhibit distinct errors associated with factors such as detector performance, interferometers design, light path, and light interaction with the samples. Spectra obtained from different sensors might require varied preprocessing methods even when analyzing the same sample. The application of identical preprocessing techniques may not necessarily lead to a substantial improvement in error correction for various instruments. Comparing the results obtained for K indices with different preprocessing techniques, the first derivative outperforms SNV but remains substantially distant from the ideal. Additionally, analysis of the image histograms reveals that the distribution on the correlation surface deviates significantly from the ideal. Moreover, upon examining the various diagonals of the covariance matrices, the substantial error associated with wavelengths around 1400 nm becomes quite apparent.

**Figure 6:**
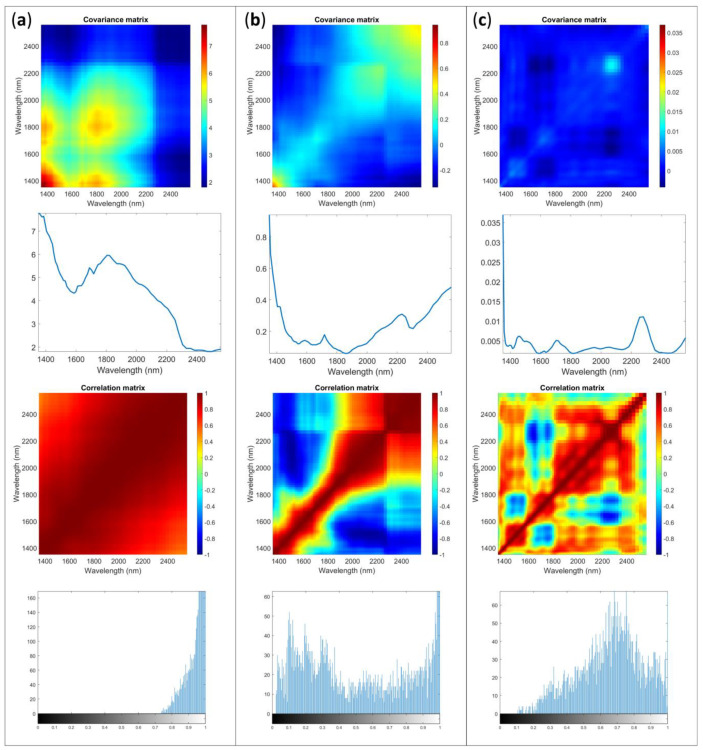
Multivariate error covariance matrix, error covariance matrix diagonal, correlation matrix and image histogram of the correlation matrix for Sample 2 acquired with NeoSpectra Scanner: (**a**) raw data (**b**) SNV (**c**) first derivative.

Furthermore, interesting results were obtained by comparing errors across different samples. In Figure 6, the errors obtained for Sample 2 are presented. It is noteworthy how the presence of the blister significantly influences the absolute error magnitude more than the shape, as also the spectral reflection (Figure 1). The applied pretreatments enable the removal of some scattering contributions but also accentuate errors in specific areas, possibly attributable to the blister plastic. In the Supplementary Materials, Figures S4 and S5 display those for Samples 3 and 4.

## 3. Materials and Methods

### 3.1. Samples

Four different samples were purchased from a local pharmacy in Como (Italy): a blue pill used as antiseptic drug, the same lot pill covered by the blister and two dietary supplements.

For clarity, through the article they are referred as Sample 1 (compact blue pill), Sample 2 (compact blue pill in the blister), Sample 3 (white compact opaque tablet), and 4 (whitish compact opaque tablet with homogenously dispersed colored particles). Samples were stored and maintained at room temperature in a protected environment (sealed containers) for the duration of the experiments. No pretreatments were conducted on the samples.

### 3.2. Spectrometers and Experiments

Two miniaturized NIR spectrometers with different technologies were used to acquire spectra considering different influencing factors of analysis. AvaSpec-Mini-NIR (Avantes, Apeldoorn, The Netherlands) was used in two different configurations: coupled with both AvaLight-HAL-S-Mini2 source and a reflection fiber probe (7 × 400 µm fibers, 2 m length, SMA term) and with AvaSphere-50-LS-HAL. NeoSpectra Scanner (Si-Ware Systems, Menlo Park, CA, USA) was used as it is, without accessories.

The different samples were acquired during the same analytical session and through repeated sessions. In particular, the factors considered were (1) the order of replicate (1 to 15), (2) the session of analysis (three independent analytical sessions), (3) the power supply during spectra acquisition (mains-connected or not, according to sensors characteristics) and (4) the timing of background. Two background timings were considered. In one case, the background was acquired before each sample along a singular independent session. In the other the background was taken only at the beginning of each analytical session.

#### 3.2.1. AvaSpec-Mini-NIR

Acquisition parameters were configured with an integration time of 15 ms and 10 averaged scans. The spectroscopic range covered was 972–1701 nm. Each spectrum resulted in 236 variables. The spectrometer was calibrated in two steps: with a black reference with the source turned off, and with a total reflectance reference using a white standard (WS-2).

For each accessory fifteen experimental replicates × 4 different samples × 3 independent analytical sessions × 2 timing of background were acquired. A total of 360 spectra was obtained for each instrumental configuration. The analysis using the optical fiber involved positioning the reflectance probe in a holder (RPH-1) and placing samples beneath it. Data were saved in .CSV format and directly imported into MATLAB for elaboration.

#### 3.2.2. NeoSpectra Scanner

A total of 720 spectra were acquired by the handheld NIR spectrometer (18.5 × 4.5 × 8 mm). Time scans of 5 s of without data interpolation were used. The spectroscopic range covered was 1351–2559 nm. The resulting spectra were composed by 74 individual variables, each. Spectra were collected after calibration using a 100% reflectance reference with a Spectralon^®^ standard, approximately 15 min after the spectrometer was powered on. Samples were directly placed over the spectrophotometer window, while the sensor was positioned with the window facing upward. The data were saved in the default “.Spectrum” format, later converted to .txt files, and processed using MATLAB R2021a.

### 3.3. Chemometrics Analysis

Spectra collected were considered as independent dataset according to instruments and samples: twelve datasets were obtained (4 for each instrumental configuration). Data mean-centering was set as default preprocessing in data analysis. Spectra visualization and Principal Component Analysis (PCA) [22] were used to identify gross errors. The spectra identified as outliers were removed from further analyses.

ANOVA–Simultaneous Component Analysis (ASCA) [23,24,25,26] was computed on the data to obtain hints on how to pursuit the following calculations on the uncertainty of data. Interpreted conceptually, this method combines analysis of variance (ANOVA) and principal component analysis (PCA) with specific constraints. ASCA approach involves decomposing the original data matrix into matrices corresponding to design factors and their interactions, which are then analyzed using simultaneous component analysis (SCA). The importance of factors and interactions is evaluated using the sum-of-squares of the corresponding submatrices. The magnitude of the calculated effect indicates the influence of the specific factor or interaction on the data. The residuals represent the unexplained variance. General information about the basics concepts of the calculation performed could be find at [27,28].

Models for each instrument and sample were calculated by evaluating different preprocessing. Two-way interactions were also calculated. A total of 2000 permutations were used to evaluate the significance of the factors: if the *p*-value was obtained < 0.05 then, the tested effect was assumed to be significant. The preprocessing methods evaluated were standard normal variate (SNV) and first order Savitzky–Golay derivative with a window width of 7 and a polynomial order of 2 [29]. Multiplicative scatter correction was evaluated in a preliminary study, but the results did not really differ from those obtained with SNV and so, they are not here reported.

Multivariate measurement error was estimated from the experimental replicates as proposed by Leger et al. [1,7]. The difference between each spectrum and the mean of the replicates was performed to obtain the error matrix. The variance-covariance (**Σcov**–also called covariance matrix) and correlation (**Σcor**) matrices were calculated for all the samples and for the different instruments. Within each instrument the errors were pooled according to the factors emerging as significant during the ASCA analysis. **Σcov** and **Σcor** result in matrices that could be visualized as images. **Σcov** provides information about the type of errors in the dataset and their reciprocal magnitude. It is dependent on the reflectance values of the spectra. The diagonal of the variance-covariance matrix provides insights into the uniformity of errors in the spectra, with uniform values indicating homoscedastic errors along the spectra. Non-uniform values, on the other hand, suggest varying errors and so, heteroscedasticity. Off-diagonal elements offer details about the covariance of measurement errors.

**Σcor** indicates the structure of the relationship among errors independently from the scale and is a matrix containing numbers ranging between −1 and 1. While the covariance matrix reveals the strength of relationships among errors, the correlation matrix, derived from it, illustrates the underlying structure of these relationships, offering complementary insights. A K index [30] is a redundancy index that could be used to resume the correlation of a set of multivariate data was calculated from the correlation matrix. Theoretically, the K index ranges from 0 to 1 and it takes its lowest value when all the variables are uncorrelated and the highest when they are correlated. For a given matrix with the number of columns greater than the number of rows, the K index can take on a minimum value equal to the imbedded correlation which depends on the dimension of the matrix. Being $\lambda_{1}$, $\lambda_{2}$, …, $\lambda_{p}$ the set of the p eigenvalues obtained from PCA applied to the correlation matrix of a dataset. The general formula for *K* index is:

(1) $$K=\frac{\overset{p}{\underset{m=1}{\sum }}\left|EV_{m}-\frac{1}{p}\right|}{\frac{2\left(p-1\right)}{p}}$$

where *EV* is the explained variance from the *m*-th principal component and could be obtained from:

(2) $$EV_{m}=\frac{\lambda_{m}}{\sum_{m=1}^{p}\lambda_{m}}$$

If considering correlation error matrices plot as digital image the number of pixel for the same instrument is always the same. According to this, comparison of the influence of preprocessing were evaluated through image histograms. An image histogram is a gray-scale chart that easily and visually shows the distribution of intensity and so the frequency of occurrence of each gray-level value. [31]. It has two axes: the x-axis represents the total number of gray levels ranging from 0 to 1 in which an image could be converted, while the y-axis represents the total number of pixels. Respectively, in this study, values around 0 correspond to values around −1 in the error correlation matrices, the 0.5 in the x of the image histogram correspond to values around 0 in **Σcor**, and value around 1 in the image histogram correspond to the ones in **Σcor**.

Data analysis was performed using routines and toolboxes developed in the MATLAB R2021a environment (the Mathworks Inc., Natick, MA, USA). Principal component analysis and ASCA were carried out by PLS-Toolbox v. 9.0 (Eigenvector Inc., Manson, WA, USA).

## 4. Conclusions

This study fills a critical gap in the current understanding of measurement errors associated with miniaturized near-infrared instruments and highlights the importance of accurate data analysis to obtain reliable scientific results. The proposed statistical methodology enables the study of data and experiments conducted with portable instrumentation. The optimal conditions for each application will depend on the researcher’s specific goals. This study facilitates an understanding of how to analyze measurement errors based on the available tools and the samples to be examined, aiming for optimization. The presented results are grounded in the concept that it is not only crucial to select the right instrument but, more importantly, to identify the best way to utilize the available tools while acknowledging their associated uncertainties. The research delves into the evaluation of multivariate measurement errors and investigates their complexities in the context of diverse samples, miniaturized NIR instruments and data preprocessing. The use of ASCA has proven to be a powerful tool for understanding how to account for experimental factors in representing multivariate measurement errors. The results highlight the possibilities and limitations of ASCA, paving the way for its effective application in similar studies. The introduction of the K index in combination with visual representations such as image histograms provides a new approach to evaluate the impact of preprocessing methods on multivariate errors. This combined approach not only provides a quantitative measure of error, but also provides an immediate visual understanding of how different preprocessing techniques affect data accuracy. By discerning which preprocessing methods bring the data closest to theoretical ideality, researchers can optimize their analytical procedures for enhanced precision and reliability. Furthermore, the samples examined in this study are sufficiently representative of possible scenarios in the pharmaceutical field, thus the results can provide interesting insights for specific studies.

## Figures and Tables

**Table 1:** Summary of results for ASCA models of AvaSpec-Mini-NIR data obtained with integrating sphere. The effects are expressed in percentage of contribution to the sum of squares of the data. Models calculated on each sample. Factors that shown a *p*-value < 0.05 after 2000 permutations are reported in bold. None is used to identify the default preprocessing. SNV = Standard Normal Variate. First derivative = Savitzky–Golay first derivative filter with 7 width and second polynomial order.

	Preprocessing	Sample	Integrating Sphere						
			Factors			Interactions			Residuals
			Order of Replicates	Session	Timing of Background	Order of Replicates × Session	Order of Replicates × Timing of Background	Session × Timing of Background	
**Effect (percentage contribution to the sum of squares)**	**None**	Sample 1	16.15	2.70	**4.97**	25.10	15.09	2.15	33.84
		Sample 2	13.02	**15.61**	**4.61**	30.30	9.00	**7.72**	19.74
		Sample 3	6.20	**42.58**	**14.08**	15.00	5.48	**5.81**	10.85
		Sample 4	11.61	**8.17**	**8.33**	17.24	7.18	**34.5176**	12.95
	**SNV**	Sample 1	6.74	**10.30**	**8.83**	22.07	19.81	**6.32**	25.92
		Sample 2	6.12	**27.68**	**28.29**	**12.16**	3.23	**15.84**	6.69
		Sample 3	2.35	**35.71**	**6.96**	5.89	2.21	**41.54**	5.33
		Sample 4	4.46	**16.27**	**16.13**	9.17	4.01	**41.71**	8.25
	**First derivative**	Sample 1	11.34	**10.87**	**15.10**	20.25	11.55	**8.25**	22.63
		Sample 2	8.81	**16.59**	**18.60**	**18.87**	5.19	**21.83**	10.11
		Sample 3	4.75	**27.55**	**4.63**	**10.66**	4.21	**40.39**	7.81
		Sample 4	5.06	**14.48**	**19.77**	11.58	5.04	**33.94**	10.13

**Table 2:** Summary of results for ASCA models of AvaSpec-Mini-NIR data obtained with optical fiber. The effects are expressed in percentage of contribution to the sum of squares of the data. Models calculated on each sample. Factors that shown a *p*-value < 0.05 after 2000 permutations are reported in bold. None is used to identify the default preprocessing. SNV = Standard Normal Variate. First derivative = Savitzky–Golay first derivative filter with 7 width and second polynomial order.

	Preprocessing	Sample	Optical Fiber						
			Factors			Interactions			Residuals
			Order of Replicates	Session	Timing of Background	Order of Replicates × Session	Order of Replicates × Timing of Background	Session × Timing of Background	
**Effect (percentage contribution to the sum of squares)**	**None**	Sample 1	10.07	2.94	0.56	30.43	17.44	**8.28**	30.28
		Sample 2	25.73	3.41	1.65	**39.52**	12.83	0.78	16.08
		Sample 3	12.50	**13.45**	**21.98**	19.16	10.41	3.25	19.26
		Sample 4	16.37	4.36	1.76	35.83	13.46	3.77	24.45
	**SNV**	Sample 1	8.52	**13.03**	**8.52**	17.48	10.06	**23.53**	18.87
		Sample 2	25.73	**3.41**	**1.65**	39.52	12.83	**0.78**	16.08
		Sample 3	7.71	3.40	**17.58**	20.98	10.66	**15.25**	24.42
		Sample 4	15.76	**6.64**	**8.44**	19.68	17.02	4.05	28.41
	**First** **derivative**	Sample 1	6.61	**27.31**	**19.27**	11.45	5.13	**20.02**	10.21
		Sample 2	25.73	**3.41**	**1.65**	39.52	12.83	**0.78**	16.08
		Sample 3	6.66	**5.13**	**20.35**	22.60	7.99	**13.01**	24.26
		Sample 4	16.59	**7.47**	**3.45**	19.56	18.65	2.34	31.93

**Table 3:** Summary of results for ASCA models of Neospectra Scanner data. The effects are expressed as percentage of contribution to the sum of squares of the data. Models calculated on each sample. Factors that shown a *p*-value < 0.05 after 2000 permutations are reported in bold. None is used to identify the default preprocessing. SNV = Standard Normal Variate. First derivative = Savitzky–Golay first derivative filter with 7 width and second polynomial order.

	Preprocessing	Sample	Factors				Interactions						Residuals
			Order of Replicates	Session	Power Supply	Timing of Background	Order of Replicates × Session	Order of Replicates × Power Supply	Order of Replicates × Timing of Background	Session × Power Supply	Session × Timing of Background	Power Supply × Timing of Background	
**Effect (percentage contribution to the sum of squares)**	**None**	Sample 1	6.67	**9.61**	0.47	0.58	14.62	6.89	6.27	1.03	**3.56**	**3.36**	46.94
		Sample 2	4.97	1.43	0.59	**10.97**	12.47	3.60	6.44	**6.29**	**6.79**	**14.58**	31.84
		Sample 3	3.05	**19.64**	**11.13**	**10.28**	7.27	1.97	2.33	**1.62**	**2.83**	**10.98**	28.93
		Sample 4	1.07	2.31	15.76	6.60	0.85	0.76	1.25	16.56	22.75	3.12	28.98
	**SNV**	Sample 1	5.22	**8.91**	**3.17**	**1.54**	15.07	7.93	4.74	**2.39**	**4.67**	**1.19**	45.17
		Sample 2	4.91	**4.79**	**3.59**	**2.15**	8.91	3.69	3.61	**6.94**	**7.49**	**9.81**	44.10
		Sample 3	5.31	**10.99**	**17.67**	**16.42**	10.99	1.44	1.93	**1.74**	**2.51**	**3.39**	27.59
		Sample 4	0.70	2.32	6.65	4.33	1.04	0.50	0.61	19.59	22.10	4.87	37.28
	**First** **derivative**	Sample 1	7.13	**3.23**	0.72	0.40	15.99	6.30	7.51	1.89	**4.63**	0.48	51.72
		Sample 2	5.02	**3.30**	**4.66**	**7.54**	10.01	4.74	4.70	**4.11**	**5.83**	**10.32**	39.77
		Sample 3	6.02	**8.33**	**16.21**	**16.48**	**10.48**	1.93	2.88	**3.54**	**3.87**	**5.30**	24.97
		Sample 4	0.88	3.36	8.19	7.35	1.71	1.00	0.88	17.62	18.30	8.40	32.32

**Table 4:** K index obtained for AvaSpec-Mini-NIR data acquired with background before each sample. SNV = Standard Normal Variate. First derivative = Savitzky–Golay first derivative filter with 7 width and second polynomial order.

	Integrating Sphere			Optical Fiber		
Preprocessing	None	SNV	First Derivative	None	SNV	First Derivative
Sample 1	0.972	0.864	0.837	0.995	0.854	0.840
Sample 2	0.985	0.873	0.848	0.999	0.917	0.891
Sample 3	0.982	0.916	0.863	0.986	0.968	0.950
Sample 4	0.979	0.942	0.896	0.984	0.971	0.957

**Table 5:** K index obtained for NeoSpectra Scanner data acquired with background before each sample and using the instrument on its own battery. SNV = Standard Normal Variate. First derivative = Savitzky–Golay first derivative filter with 7 width and second polynomial order.

	NeoSpectra Scanner		
Preprocessing	None	SNV	First Derivative
Sample 1	0.94	0.94	0.82
Sample 2	0.97	0.94	0.88
Sample 3	0.90	0.86	0.88
Sample 4	0.89	0.83	0.80

## Data Availability

The data presented in this study are available on request from the corresponding author. The data are not publicly available due to authors’ decision.

## Supplementary Materials

The following supporting information can be downloaded at: https://www.mdpi.com/article/10.3390/molecules28247999/s1.
