# Supplementary material for: A Multivariate Analysis-Driven Workflow to Tackle Uncertainties in Miniaturized NIR Data
Source: Molecules. 2023 Dec 7;28(24):7999. doi: 10.3390/molecules28247999 (PMC10745448; doi:10.3390/molecules28247999)
Supplement: Supplementary file 1 [file molecules-28-07999-s001.zip › molecules-2748054-supplementary.pdf]

A Multivariate Analysis-Driven Workflow to Tackle Uncertainties in Miniaturized NIR Data—Supplementary Materials

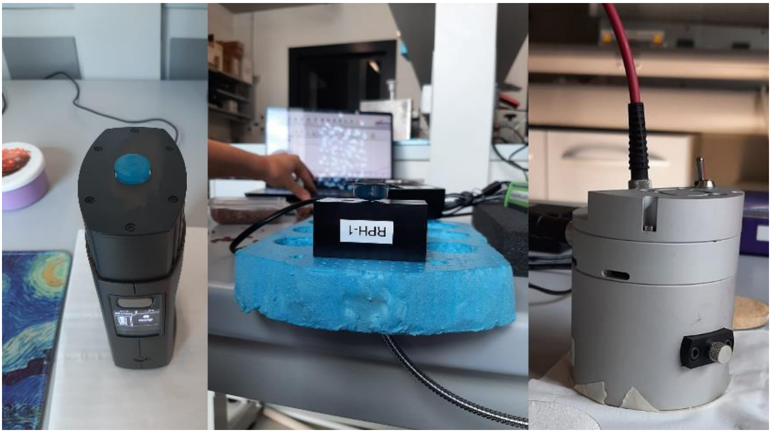

Figure S1. Example of configuration for spectral acquisitions.

Table S1. Descriptive statistics of spectra acquired with AvaSpec-Mini-NIR. Calculated on percentage reflectance data.

	**Integrating sphere**				**Optical fiber**			
	**Sample 1**	**Sample 2**	**Sample 3**	**Sample 4**	**Sample 1**	**Sample 2**	**Sample 3**	**Sample 4**
**Relative standard deviation (%) – mean**	6.06	3.96	2.20	1.57	126.99	73.39	9.42	10.05
**RMS – mean**	0.51	0.53	1.02	0.95	0.61	12.18	3.81	3.67
**RMS – standard deviation**	0.86	0.28	0.51	0.34	0.88	10.28	2.48	2.57
**S/N mean**	8.74	25.96	57.49	92.14	0.98	1.37	11.18	10.34
**S/N standard deviation**	0.33	4.38	25.29	46.31	0.41	0.07	2.24	1.87

Table S2. Descriptive statistics of spectra acquired with NeoSpectra Scanner. Calculated on percentage reflectance data.

	**Sample 1**	**Sample 2**	**Sample 3**	**Sample 4**
**Relative stand deviation (%) – mean**	17.39	30.09	2.36	2.51
**RMS – mean**	0.41	2.36	0.78	1.26
**RMS – standard deviation**	0.25	1.29	0.36	0.37
**S/N mean**	5.98	3.44	65.05	47.02
**S/N standard deviation**	1.21	0.71	44.10	18.05

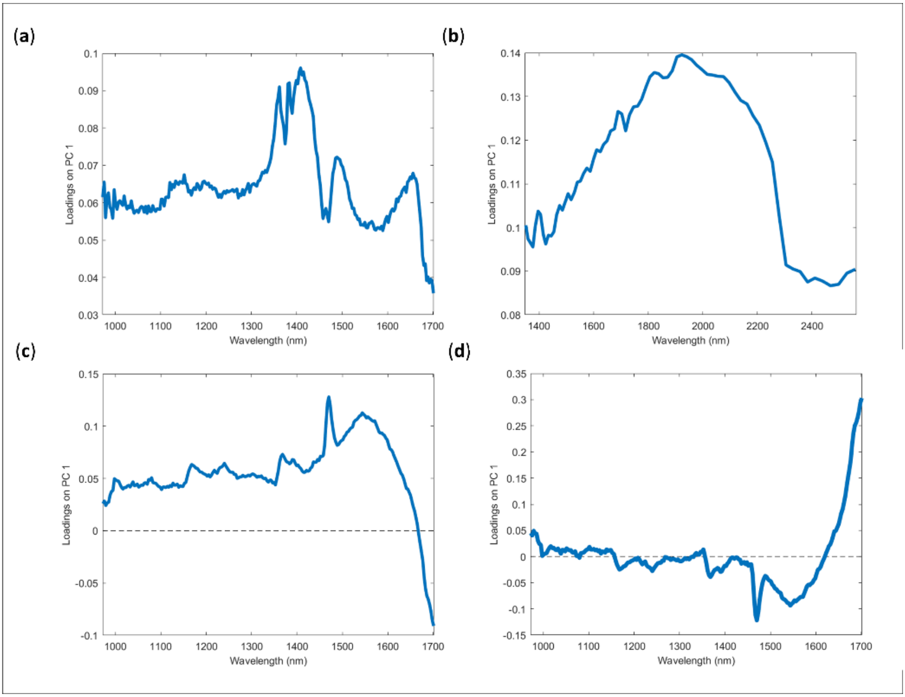

Figure S2. ASCA submodels examples. Loadings of the ASCA sub–model for the factor (a) session (b) replicates (c) and (d) timing of background. Instruments: (a) AvaSpec-Mini-NIR equipped with integrating sphere (b) NeoSpectra Scanner (c) and (d) AvaSpec-Mini-NIR equipped with optical fiber. Samples: (a) Sample 3 (b) Sample 2 (c) and (d) Sample 4.

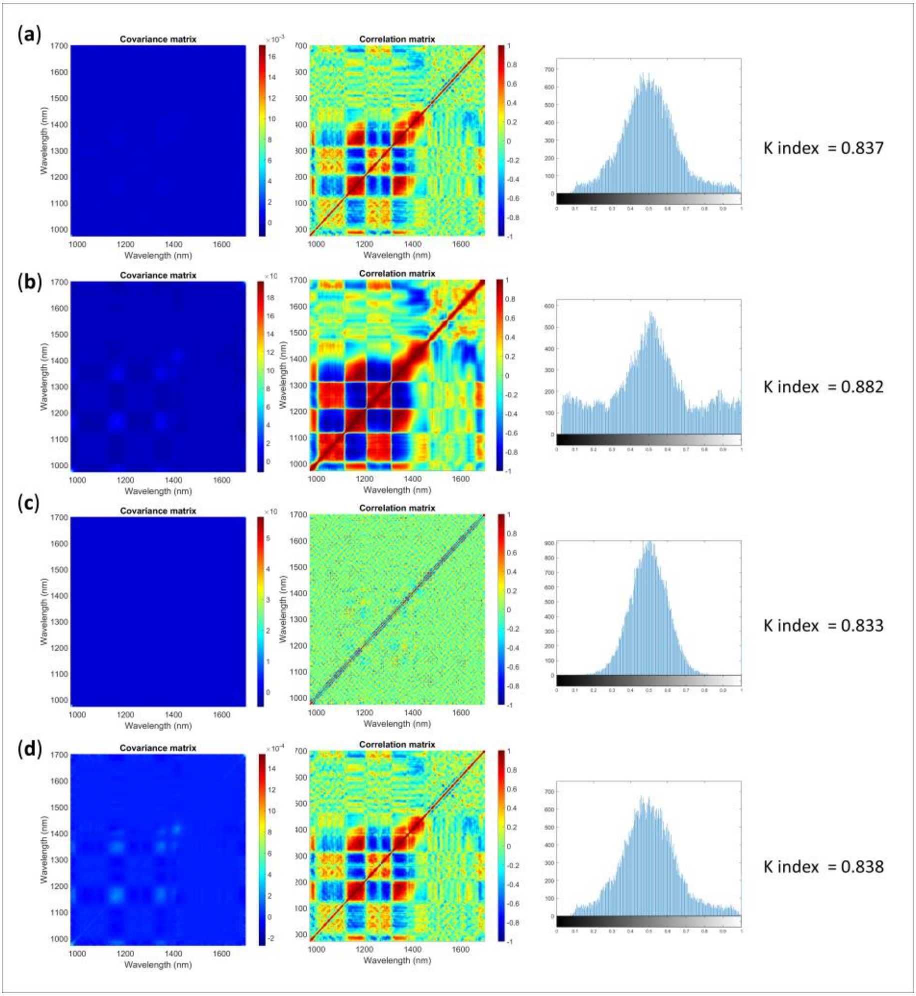

Figure S3. Multivariate error covariance matrix, correlation matrix, image histogram and K index for Sample 1 acquired with AvaSpec-Mini NIR with integrating sphere and preprocessed with first derivative (a) w = 7, d=1 ,p=2; (b) w = 15, d=1 ,p=2 ; (c) w = 7, d=2 ,p=2; (d) w = 7, d=1 ,p=1.

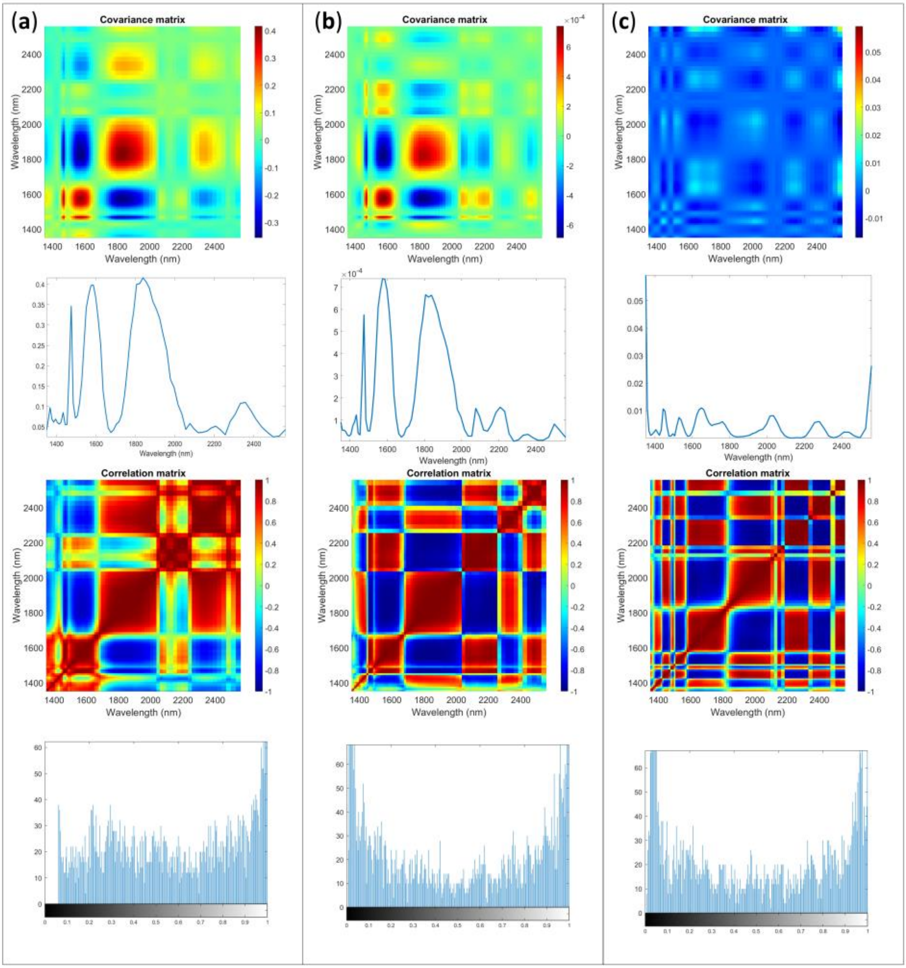

Figure S4. Multivariate error covariance matrix, error covariance matrix diagonal, correlation matrix and image histogram of the correlation matrix for Sample 3 acquired with NeoSpectra Scanner: (a) raw data (b) SNV (c) first derivative.

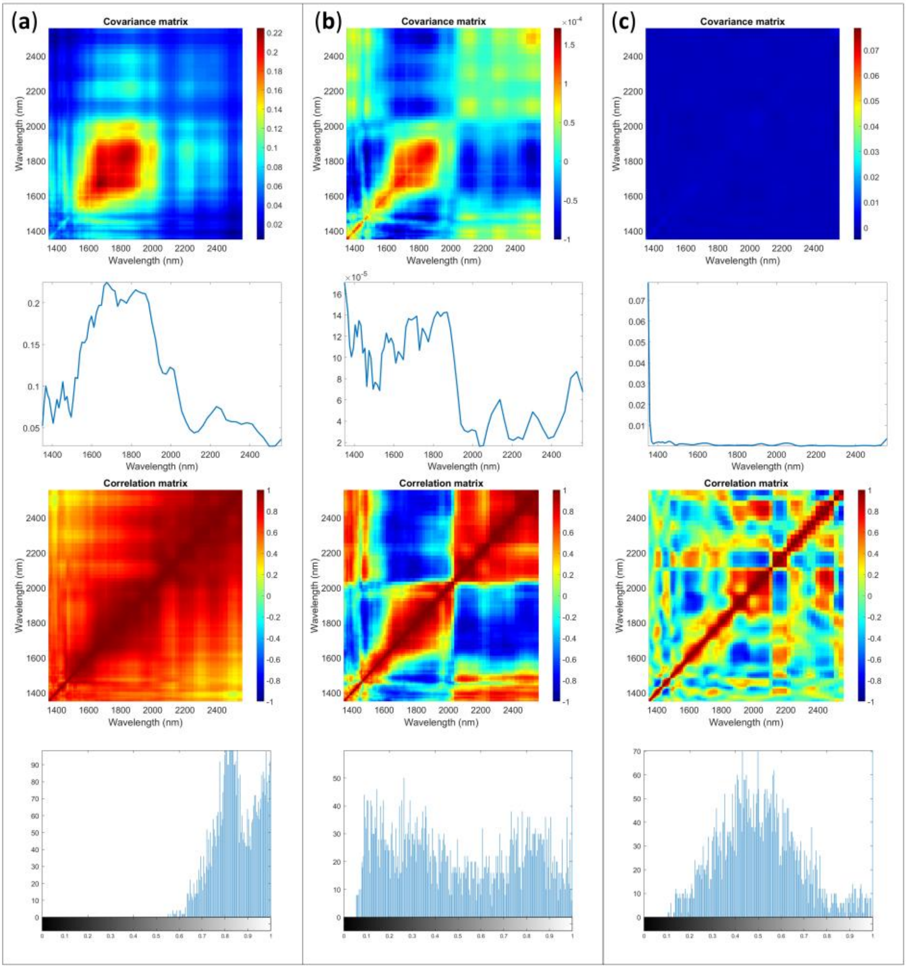

Figure S5. Multivariate error covariance matrix, error covariance matrix diagonal, correlation matrix and image histogram of the correlation matrix for Sample 4 acquired with NeoSpectra Scanner: (a) raw data (b) SNV (c) first derivative.
